# Diverging Trends in Cause-Specific Mortality and Life Years Lost by Educational Attainment: Evidence from United States Vital Statistics Data, 1990-2010

**DOI:** 10.1371/journal.pone.0163412

**Published:** 2016-10-04

**Authors:** Isaac Sasson

**Affiliations:** Department of Sociology and Anthropology, Tel Aviv University, Tel Aviv 6997801, Israel; St. Michael's Hospital, CANADA

## Abstract

**Background:**

Life expectancy at birth in the United States will likely surpass 80 years in the coming decade. Yet recent studies suggest that longevity gains are unevenly shared across age and socioeconomic groups. First, mortality in midlife has risen among non-Hispanic whites. Second, low-educated whites have suffered stalls (men) or declines (women) in adult life expectancy, which is significantly lower than among their college-educated counterparts. Estimating the number of life years lost or gained by age and cause of death, broken down by educational attainment, is crucial in identifying vulnerable populations.

**Methods and Findings:**

Using U.S. vital statistics data from 1990 to 2010, this study decomposes the change in life expectancy at age 25 by age and cause of death across educational attainment groups, broken down by race and gender. The findings reveal that mortality in midlife increased for white women (and to a lesser extent men) with 12 or fewer years of schooling, accounting for most of the stalls or declines in adult life expectancy observed in those groups. Among blacks, mortality declined in nearly all age and educational attainment groups. Although an educational gradient was found across multiple causes of death, between 60 and 80 percent of the gap in adult life expectancy was explained by cardiovascular diseases, smoking-related diseases, and external causes of death. Furthermore, the number of life years lost to smoking-related, external, and other causes of death increased among low- and high school-educated whites, explaining recent stalls or declines in longevity.

**Conclusions:**

Large segments of the American population—particularly low- and high school-educated whites under age 55—are diverging from their college-educated counterparts and losing additional years of life to smoking-related diseases and external causes of death. If this trend continues, old-age mortality may also increase for these birth cohorts in the coming decades.

## Introduction

Life expectancy at birth in the United States is currently estimated at 78.7 years for men and women combined—a 31-year increase since 1900—and is projected to surpass 80 years within the next decade [1,2]. At the same time, socioeconomic disparities in adult mortality, especially when measured via educational attainment, are greater than ever. The lifespans of low-educated Americans are shorter, on average, and exhibit more variability than those of their college-educated counterparts [3–9]. Low-educated whites in particular have been subjected to increasing mortality rates in midlife [10] and, at least among women, also significant declines in adult life expectancy [11–13]. As a result, the education gap in adult life expectancy—the difference between those with college education or higher and those with fewer than 12 years of schooling—increased more than threefold among white women (2.5 to 9.3 years) and nearly doubled among white men (6.1 to 11.9 years) between 1990 and 2010 [13]. Taken together, these studies question the presumption that life expectancy will continue to rise for all population groups in the United States or for the country as a whole. Indeed, such a sustained decline in life expectancy is unprecedented in high-income countries [10].

However, there are considerable ongoing debates regarding the extent to which life expectancy declined among the least educated white men and women—those with fewer than 12 years of schooling—depending on the source of data used and categorization of educational attainment groups. Using vital registry and census data, Olshansky and colleagues [12] reported a decline of 5.3 and 3.4 years in life expectancy at age 25 among low-educated, non-Hispanic white women and men, respectively, between 1990 and 2008. Using similar data, Sasson [13] provided more conservative estimates of the same decline in adult life expectancy from 1990 to 2010–3.1 years for women and 0.6 years for men. The latter are more consistent with estimates derived from the National Health Interview Survey (NHIS) from 1990 to 2005, showing a 3.2-year decline among low-educated white women and a 0.9-year *increase* among men [11]. Emphasizing relative, rather than fixed, levels of education, Bound and colleagues [14] found a 1.2-years decline in the lowest quartile of educational attainment among white women and a 0.4-year increase among white men between 1990 and 2010 using vital registry data. In spite of discrepancies between different data sources and approaches to measuring educational attainment, even the most conservative estimates reflect an excess of nearly 170,000 deaths among low-educated white women due to rising mortality between 1990 and 2010 [15].

Both survey data and the vital registry have their advantages and shortcomings in estimating socioeconomic disparities in mortality. Census-unlinked data tend to overestimate disparities in adult mortality [16] and, particularly in the United States, are less reliable with respect to education reporting [17,18]. By contrast, education is self-reported in the NHIS and therefore more reliable. At the same time, NHIS data are limited to the non-institutionalized population and linkage with death records is imperfect, which underestimates mortality in general and possibly disparities between groups [19].

Surprisingly, little is currently known about the contribution of specific causes of death to the widening education gap in adult life expectancy or the absolute decline observed among low-educated white Americans. Although several studies have documented trends in cause-specific mortality or relative risks by educational attainment [20–22], none have translated those differences into life years lost—with the exception of cigarette smoking [23]—a necessary step in identifying the primary risk factors and where policy intervention can be most effective in reducing disparities in longevity. Indeed, educational differences in mortality are observed in nearly all causes of death [21] and have grown significantly over the past two decades, especially for heart disease, cancer, and smoking-related diseases [7, 20].

For some causes of death, mortality declined across all education groups, although greater declines were observed among the college-educated, whereas for other causes mortality declined among the highly educated but increased among the low educated. For example, between 1986 and 2006 mortality rates among white women aged 45–84 with fewer than 12 years of schooling *increased* for lung cancer, cerebrovascular diseases, chronic lower respiratory diseases, diabetes, and Alzheimer’s disease [22]. These women were five times more likely to die of cardiovascular diseases and ten times more likely to die of influenza and pneumonia than their college-educated counterparts. Yet, the direct impact of those relative risks on the education gap in life expectancy—let alone for other gender and race groups—remains unknown. A notable exception is the case of cigarette smoking. Ho and Fenelon [23] found that while educational disparities in life expectancy at age 50, $e_{50}^{o}$, have been increasing for non-Hispanic whites over time, the proportion of the education gap attributed to smoking has declined (especially for men). For example, in the 1980s the gap in $e_{50}^{o}$ between college-educated white men and those with high-school education or less amounted to 4.3 years—44.3 percent of which was attributed to smoking. By 2003–2006, the same gap increased to 5.4 years, but only 33.8 percent of which was due to smoking. However, their analysis was limited to smoking and did not account of other causes of death.

Using U.S. vital registry data from 1990 to 2010, this study aims to reveal (a) across educational attainment groups, in which age groups gains (losses) in life expectancy at age 25 have been most pronounced; (b) which causes of death account for recent gains (declines) in adult life expectancy, by educational attainment (within-group trends); and (c) which causes of death account for the widening education gap in adult life expectancy (between-group trends). I focus in particular on non-Hispanic whites (hereafter whites) because they recently experienced rising mortality in midlife and exhibit the largest education gap in longevity. Nevertheless, results for non-Hispanic blacks are shown in the Supporting Information (S3 Appendix). Relying on the vital registry provides the level of detail necessary to extend the analysis beyond most survey data to multiple populations, age groups, and specific causes of death. However, these data are not without limitations. By addressing data quality issues in the methods section, the present study is able to provide reliable, nationally representative estimates of the education gap in mortality and change therein by age and cause of death.

## Methods

### Data

I estimated age-cause-specific mortality rates, by race, gender, and educational attainment, from two data sources. Death counts were obtained from the 1990, 2000, and 2010 Multiple Cause of Death (MCD) public use data files [24], which include information from all death certificates issued in the United States in a given year. Person-years of exposure were based on midyear population estimates from the 5% Integrated Public Use Microdata Sample [25] in respective census years. The analysis was limited to non-Hispanic whites and blacks (results for the latter are shown in the Supporting Information) because Hispanic origins are more often misclassified on death certificates [26]. I focus in particular on mortality between ages 25 and 85 because educational attainment at the college level is generally completed by age 25, and because age and cause of death reporting in vital registries are less reliable among the oldest old [27–29]. Nevertheless, the results confirm that much of the education gap in life expectancy at age 25, $e_{25}^{o}$, is captured in this age range.

Educational attainment was recoded into four categories based on completed years of schooling: low (0–11), high school (12), some college (13–15), and college degree or higher (16+). However, estimating educational disparities in mortality using unlinked data (i.e., where the numerator and denominator come from different data sources) is especially prone to bias [16]. Whereas the census data is generally complete, education reporting on U.S. death certificates is often inaccurate or missing altogether [17]—particularly in earlier years and among older decedents [18]. Beginning with the 2003 revision of the U.S. death certificate, educational attainment was measured in grades/degree categories rather than single years. By 2010, the new version has been adopted by 34 states and the District of Columbia [30]. I translated education categories to completed years of schooling in order to ensure consistency over time. Most importantly, deaths in the "High school graduate or GED completed" category were recoded as 12 years of schooling; deaths in the "9th—12th grade, no diploma" category were reallocated to 9–11 and 12 years of schooling, respectively, proportional to their relative size in the 2010 American Community Survey. This approach is rather conservative, because it assumes an equal risk of mortality in those groups. In addition, I imputed missing data on educational attainment using a simple Bayesian model by age group, race, gender, and state of occurrence, pooling information from both the vital registry and census data. The measures taken to ensure data quality and consistency over time, as well as missing data imputation, are discussed extensively elsewhere [13].

In addition to information on the decedent’s race, gender, age at death, and educational attainment, the MCD includes an underlying a cause of death code based on the International Classification of Diseases (ICD) [31], using the 9^th^ revision in 1990 and the 10^th^ revision in 2000 and 2010. I grouped all causes of death in nine major categories in order to ensure consistency throughout the study period and to avoid cells with small death counts (see Supporting Information, S1 Appendix for a complete list of codes). The nine categories include: infectious and parasitic diseases, neoplasms (excluding those predominantly attributed to smoking), cardiovascular diseases (CVD), cerebrovascular diseases, smoking-related diseases (SRD), respiratory diseases (excluding chronic lower respiratory diseases), diabetes mellitus, external causes, and a residual category for all other or unspecified causes. SRDs were defined as causes in which the smoking-attributable fraction of deaths exceeds 65 percent in men and women combined [32]. This category includes cancers of the lip, oral cavity, pharynx, esophagus, larynx, trachea, lung, and bronchus, as well as chronic lower respiratory diseases (bronchitis, emphysema, and chronic airway obstruction)—but omits many more deaths attributable to smoking such as ischemic heart disease, where the smoking-attributable fraction of death is 16 percent. Nevertheless, trends in SRDs as defined here most clearly reflect underlying smoking behavior over time and across subpopulations. The number of deaths from each of the nine categories by year, gender, and educational attainment is available in Supporting Information (S2 Appendix).

Using the age-cause-specific mortality rates, I constructed single- and multiple-decrement period life tables for each race, gender, and educational attainment group in 1990, 2000, and 2010. First, I decomposed the *change* in adult life expectancy by age in each of those groups. Second, I estimated trends in years of life lost to each cause of death, by educational attainment, throughout the 20-year period.

### Age Decomposition of Change in Life Expectancy

The change in life expectancy at age 25 between Time 1 and Time 2, $△e_{25}^{o}=e_{25}^{o}\left(2\right)-e_{25}^{o}\left(1\right)$ in standard life table notation, can be decomposed by age group in the following manner [33,34]:

$${\;}_{n}{△}_{x}=\frac{l_{x}^{1}}{l_{25}^{1}}\left(\frac{{\;}_{n}L_{x}^{2}}{l_{x}^{2}}-\frac{{\;}_{n}L_{x}^{1}}{l_{x}^{1}}\right)+\frac{T_{x+n}^{2}}{l_{25}^{1}}\left(\frac{l_{x}^{1}}{l_{x}^{2}}-\frac{l_{x+n}^{1}}{l_{x+n}^{2}}\right)$$ (1)

where _n_Δ_x_ is the gain or loss in total life expectancy attributed to the change in mortality rate between ages *x* and *x+n*, l_x_ is the number of survivors at age *x*, _n_L_x_ is the number of person-years lived between ages *x* and *x+n*, T_x+n_ is the number of person-years lived after age *x+n*, and the superscripts denote Time 1 or 2. In other words, Eq (1) shows the change in life expectancy due to additional (or fewer) survivors between ages *x* and *x+n* as the sum two components. The first term on the right-hand side, known as the direct effect, is the number of additional (fewer) years lived between ages *x* and *x+n*; the second term, combining the indirect plus interaction effects, is the additional (fewer) years lived in *subsequent* age groups. Clearly, only the direct effect applies to the open age interval:

$${\;}_{\infty }{△}_{x}=\frac{l_{x}^{1}}{l_{25}^{1}}\left(\frac{T_{x}^{2}}{l_{x}^{2}}-\frac{T_{x}^{1}}{l_{x}^{1}}\right)$$ (2)

Finally, summing the contributions across all age groups equals the total change in life expectancy at age 25 between Time 1 and Time 2:

$$△e_{25}^{o}=e_{25}^{o}\left(2\right)-e_{25}^{o}\left(1\right)=\sum_{25}^{\infty }{}_{n}{△}_{x}$$ (3)

Arriaga’s decomposition reveals how changes in age-specific mortality rates translate into gains or losses in overall life expectancy. In other words, it can reveal scenarios in which life years gained by declining mortality in certain age groups are offset by losses from increasing mortality in other age groups, as recent studies have shown [10].

### Years of Life Lost by Cause of Death

In addition to age-specific contributions to change in life expectancy, it is important to understand which causes of death explain the educational gap in life expectancy as well as within-group trends in longevity. Although there are several methods to calculate the number of life years lost to specific causes of death [34,35], most rely on unrealistic assumptions (e.g., causes of death are eliminated altogether, are independent of one another, or decline proportionally across all ages). Therefore, in this paper I adopt an alternative measure of years of life lost (YLL) based on the cumulative incidence of each cause of death [36]:

$$YLL=60{-}_{60}e_{25}$$ (4)

where *YLL* is the expected number of life years lost from all causes between ages 25 and 85 and _60_*e*_25_ is the temporary life expectancy in the same age interval. *YLL* can be further decomposed into specific causes of death using standard functions from the multiple-decrement life table. When the life table radix, *l*_25_, equals unity, _*n*_*L*_*x*_ equals the mean number of years lived by an individual between ages *x* and *x+n*. The mean number of years lost in that interval, denoted _*n*_ℸ_*x*_ as the complement of _*n*_*L*_*x*_, is therefore:

$${\;}_{n}{ℸ}_{x}=n{-}_{n}L_{x}$$ (5)

Following Andersen and colleagues [36], Eq (5) can then be decomposed into contributions from each cause of death:

$${\;}_{n}{ℸ}_{x}^{i}=n\cdot {\;}_{x}d_{25}^{i}+\left(n\cdot l_{x}{-}_{n}L_{x}\right)\cdot \frac{{\;}_{n}d_{x}^{i}}{{\;}_{n}d_{x}}$$ (6)

where ${\;}_{n}{ℸ}_{x}^{i}$ is the number of years lost due to cause *i* between ages *x* and *x+n*, *n* is the length of the age interval, *l*_*x*_ and _*n*_*d*_*x*_ are the standard life table functions, ${\;}_{x}d_{25}^{i}$ is the cumulative number of life table deaths from cause *i* by age *x* (this also equals the probability of dying from cause *i* before age *x* when *l*_25_ = 1), and $\frac{{\;}_{n}d_{x}^{i}}{{\;}_{n}d_{x}}$ is the fraction of deaths in the age interval due to cause *i*. The first term on the right-hand side of Eq (6) can be interpreted as the number of life years lost between ages *x* and *x+n* to deaths from cause *i* that occurred before age *x* (i.e., they each lose *n* years); the second term equals the number of years lost due to deaths from cause *i* during the interval (i.e., each death contributes _*n*_*a*_*x*_ lost years on average, where _*n*_*a*_*x*_ is the standard life table function). *YLL* is therefore the sum of years lost from all causes *i* across all *j* intervals of length *n*:

$$YLL=\sum_{j}\sum_{i}{}_{n}{ℸ}_{x}^{i}$$ (7)

By omitting the open interval and focusing on the temporary life expectancy between ages 25 and 85, this definition of YLL presents several advantageous properties: (a) it is based on actual number of years lost rather than on a hypothetical population; (b) the number of life years lost from each cause of death are additive to the total number of years lost; (c) it does require that competing risks are independent; and (d) it can be easily estimated from multiple-decrement life tables.

Using the two decomposition methods, the next section reveals which age groups and which causes of death were responsible for the increase or decline in adult life expectancy among low, high school, and college-educated Americans from 1990 to 2010.

## Results

### Age Decomposition of Change in Life Expectancy

**Fig 1:**
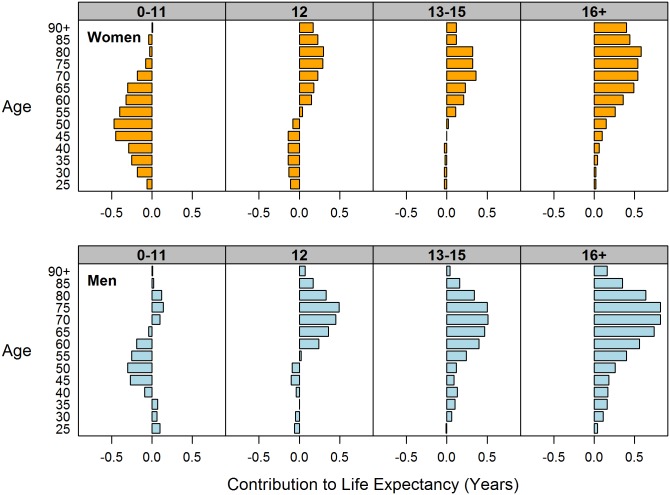
Age decomposition of change in life expectancy by gender and years of schooling, non-Hispanic whites 1990–2010.

Decomposing the change in adult life expectancy by age, within each education group, is an important first step in identifying the most vulnerable subpopulations. For example, although adult life expectancy has been declining among low-educated white Americans since 1990, especially among women, this pattern may not be equally shared across all ages. Fig 1 shows the age decomposition of change in $e_{25}^{o}$ between 1990 and 2010 for each of the educational attainment groups: low (0–11 years of schooling), high school (12), some college (13–15), and college or higher (16+). Each horizontal bar represents the contribution, in years, of changing mortality in a 5-year age group to the overall change in $e_{25}^{o}$; the sum of the bars in each column equals the total change in life expectancy.

Between 1990 and 2010, life expectancy at age 25 declined by 3.1 years among low-educated white women (Fig 1, top panel). Although mortality increased in all age groups, more than half of the decline in $e_{25}^{o}$ was attributed to rising mortality between ages 45 and 64. Therefore, targeting this vulnerable population will have the greatest impact on closing the education gap in longevity. Over the same period, life expectancy among high school—educated white women increased by less than one year because gains in longevity above age 55 were almost entirely offset by losses below that age. In other words, the modest increase in $e_{25}^{o}$ masks opposite trends among the young and the old. While older women in this group experienced further reduction in mortality, the trend has reversed for younger women. Trends among white women with some college education resemble those of their high-school educated counterparts of the same age. However, gains in life expectancy above age 55 were greater and losses below 55 were less pronounced, resulting in a net increase in $e_{25}^{o}$ of 1.5 years. Finally, college-educated white women experienced mortality decline across all age groups, amounting to a 3.7 year increase in $e_{25}^{o}$, with most gains attributed to ages 65 and over.

Overall, trends in $e_{25}^{o}$ were more favorable among white men at each level of education, but the age-specific patterns were remarkably similar to those of women (Fig 1, bottom panel). Life expectancy at age 25 among low-educated men declined by less than one year and, similar to low-educated women, was mostly due to rising mortality between ages 45 and 64. However, increasing mortality in midlife was largely offset by modest mortality declines in other age groups. Results for high-school educated white men also resembled those of women, with mortality declining significantly at ages 60 and over but slightly increasing at younger ages, resulting in a 1.8-year increase in $e_{25}^{o}$. By contrast, white men with 13–15 and 16+ years of schooling experienced morality decline across the board, with $e_{25}^{o}$ increasing by 3.1 and 5.2 years, respectively. Like their women counterparts, those gains were most pronounced at ages 60 and over.

For both men and women, in nearly all education groups, old-age mortality decline accounted for the increase in adult life expectancy from 1990 to 2010 (or, among the low-educated, for offsetting the decline in life expectancy).

### Years of Life Lost by Cause of Death across Education Groups

The temporary life expectancy between ages 25 and 85, _60_*e*_25_, is the number of years a person is expected to live during that 60-year interval. Although oldest-old mortality is truncated by design, _60_*e*_25_ closely resembles the total life expectancy at age 25, remaining about 1–2 years lower than $e_{25}^{o}$ among men and 3–4 years lower among women across educational attainment groups. Therefore, it captured much of the education gap in longevity throughout the study period. The complement of _60_*e*_25_ is the expected number of years of life lost from all-cause mortality (i.e., *YLL* = 60−_60_*e*_25_), which can be further broken down by cause of death.

**Fig 2:**
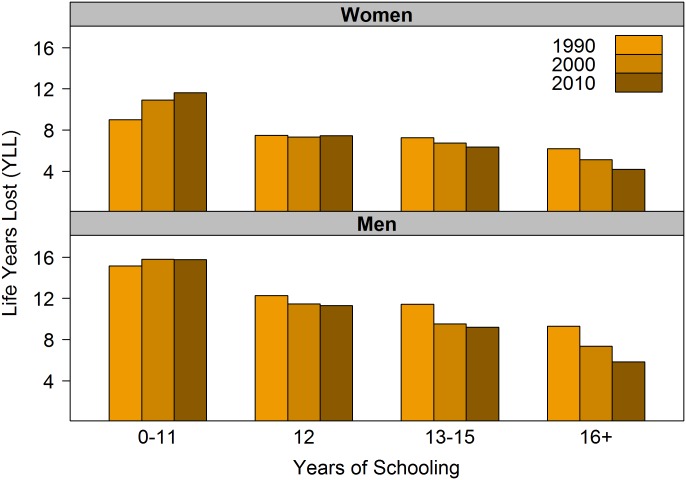
Total life years lost between ages 25 and 85 by gender and years of schooling, non-Hispanic whites 1990–2010.

Fig 2 shows the trend in YLL between ages 25 and 85, by educational attainment, from 1990 to 2010. Mirroring trends in $e_{25}^{o}$ among white women, YLL gradually increased from 9.0 years in 1990 to 11.6 years in 2010 for the low educated, stalled around 7.4 years for the high-school educated, and declined for those with 13–15 and 16+ years of schooling—from 7.3 to 6.4 and from 6.2 to 4.2, respectively. In comparison, white men lost more years of life by age 85 than their women counterparts, in all education groups, and exhibited a wider education gap in YLL. The number of years of life lost increased slightly for low-educated white men during the 1990s and plateaued during the 2000s at 15.8 years. In all other education groups, however, YLL declined for men—from 12.3 to 11.3 years among the high-school educated, from 11.4 to 9.2 years among those with some college education, and from 9.3 to 5.8 among the college educated. In both genders, the education gap in YLL—the difference between the least and most educated groups—increased over time and was greatest in 2010, reaching 7.5 years among white women and 10.0 years among white men.

**Fig 3:**
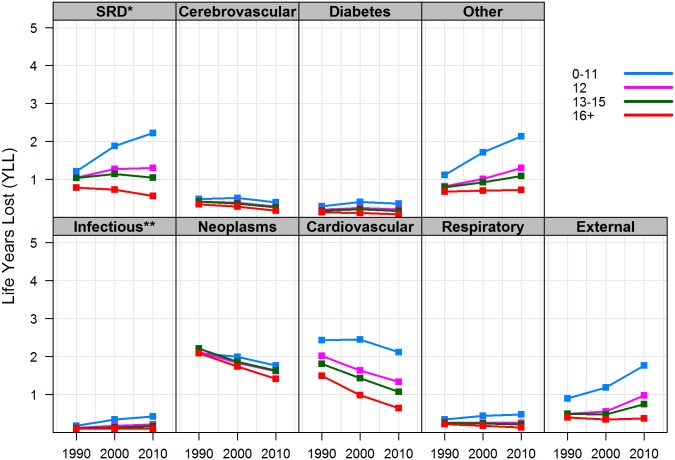
Life years lost between ages 25 and 85 by cause of death and years of schooling, non-Hispanic white women. Note: * SRD = smoking-related diseases (bronchitis, emphysema, chronic airway obstruction and cancers of the lip, oral cavity, pharynx, esophagus, larynx, trachea, lung, and bronchus); ** Infectious and parasitic diseases.

Further decomposition of YLL reveals which causes are responsible for the greatest loss of life years with education groups, as well as for gaps between them. Fig 3 shows the number of life years lost to each major cause of death, by educational attainment, among white women from 1990 to 2010. Among the low educated, YLL increased by roughly one year for smoking-related diseases, external causes, and the residual category (“other causes”). Together, these causes of death accounted for more than the total increase in YLL, but were offset by minor reductions in YLL—about one third of a year each—from CVD and neoplasms. YLL also increased for diabetes and infectious and respiratory diseases, but their combined effect was less than 0.5 additional life years lost—far below the rising toll from each of the three leading causes.

Similar trends were observed among high-school educated women, but changes in YLL from different causes of death offset each other almost entirely—i.e., increases from smoking-related diseases, external, and “other” causes were more modest while reductions from CVD and cancer were greater than among low-educated women. Surprisingly, women with some college education also experienced an increase in YLL from external and “other” causes, albeit to a lesser degree, but no significant change attributed to smoking-related diseases. Finally, college-educated women experienced reductions in YLL almost uniformly across all causes of death—the greatest of which from CVD and neoplasms, 0.7 and 0.9 years, respectively—resulting in the overall increase in adult life expectancy, temporary or total, shown earlier.

**Fig 4:**
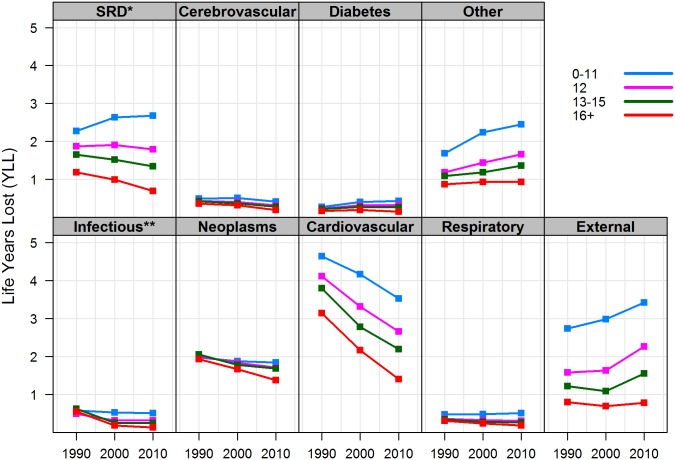
Life years lost between ages 25 and 85 by cause of death and years of schooling, non-Hispanic white men. Note: * SRD = smoking-related diseases (bronchitis, emphysema, chronic airway obstruction and cancers of the lip, oral cavity, pharynx, esophagus, larynx, trachea, lung, and bronchus); ** Infectious and parasitic diseases.

Trends among men were generally similar (but not identical) to those among women of the same educational level. Fig 4 shows that low-educated men gained more than one year of life due to reduction in CVD mortality, but experienced greater combined losses from smoking-related, external, and “other” causes. The latter two also increased among men with high-school and some college education, but declined for CVD and smoking-related diseases. Like their women counterparts, college-educated men experienced a decline in YLL in nearly all causes of death.

Overall, trends in YLL by cause of death suggest that mortality declines from CVD and neoplasms have been a success story in all gender and education groups, but have been greatest among the college educated. By contrast, YLL from external and “other” causes have been rising in almost all education groups, with the exception of college-educated men and women. In addition, mortality from smoking-related diseases increased among low-educated men and women as well as high school—educated women. Although changes in YLL were also observed in diabetes, infectious, respiratory, and cerebrovascular diseases, their combined toll on life expectancy, from a population perspective, was lower than each of the leading causes of death—CVD, neoplasms, and smoking-related diseases. By 2010, these three major causes were responsible for over 50 percent of life years lost in all gender-education groups. Among the low educated in particular, external and “other” causes also had a tremendous toll on the number of years lost, both in absolute and in relative terms. This is an important observation, because even a modest increase in the number of premature deaths among the low educated can have a significant effect on adult life expectancy and, at the same time, contribute to the growing education gap in longevity.

### The Educational Gradient in Years of Life Lost by Cause of Death

Comparing YLL by cause of death, across education groups, can also point to where disparities in life expectancy are greatest and where policy intervention may be most effective. Two general patterns can be discerned in Figs 3 and 4. First, there is a clear education gradient in YLL across all causes of death and in both genders, with more education associated with fewer life years lost. Throughout the study period, between 60 and 80 percent of the gap in _60_*e*_25_ (or, equivalently, in YLL) between low- and college-educated men and women was attributed to CVD, smoking-related diseases, and external causes of death. Although (non-smoking related) neoplasms account for a large proportion of YLL in each education group, they explain less than five percent of the education gap in _60_*e*_25_. Similarly, disparities in YLL attributed to diabetes and infectious, respiratory, and cerebrovascular diseases *combined* explain less than 20 percent of the education gap in _60_*e*_25_ in women and less than 15 percent of the gap in men. In other words, although an education gradient in YLL exists in nearly all causes of death, reducing mortality from CVD, smoking, and external causes among low education groups will have the greatest impact on closing the gap in adult life expectancy.

The second important observation is that the education gap in YLL widened across all causes of death and in both genders since 1990. For causes in which mortality declined, such as neoplasms and CVD, the college educated saw greater reductions; for most of the remaining causes of death, however, YLL increased for the low educated and declined for the college educated, in both genders. For example, in 1990, low-educated men lost 1.5 *more* years of life from CVD than college-educated men. The gap increased to 2.1 years by 2010 in spite of significant declines in CVD mortality in both groups. By contrast, men’s education gap in YLL to smoking-related diseases increased from 1.1 years in 1990 to 2.0 years in 2010—about half of which because conditions improved among the college educated and the other half because conditions worsened for the low educated. This pattern not only reflects greater health returns to higher education, but also the absolute deterioration of population health among low-educated white Americans.

## Discussion

Between 1990 and 2010, the gap in adult life expectancy between low- and college-educated white Americans has doubled for men and more than tripled for women [12,13]. This trend was fueled by both a significant rise in $e_{25}^{o}$ among the college educated and a simultaneous decline in $e_{25}^{o}$ among the low educated. Over the same period, high school—educated whites experienced only modest gains in adult life expectancy. Using vital statistics data, this study set out to understand which causes of death accounted for the widening education gap in life expectancy and in which age groups—an important step in identifying vulnerable groups and informing health policy.

Four key insights are supported by the evidence: (a) low-educated white Americans are not following in the footsteps of their college-educated counterparts and instead experienced stalling (men) or declining (women) life expectancy at age 25; (b) this trend is largely attributed to increasing morality in midlife (ages 45–64), which was also observed among the high-school educated; (c) life expectancy declined or stalled in these groups almost entirely because mortality increased for smoking-related, external, and residual (“other”) causes of death—offsetting longevity gains from declining cancer and cardiovascular mortality; and (d) significant educational differences in mortality were also observed in diabetes, infectious, respiratory, and cerebrovascular diseases, but their combined effect on the education gap in life expectancy was smaller than that of smoking or cardiovascular diseases or external causes alone.

Although midlife mortality is on the rise for low- and high school-educated whites, one should be reminded that these estimates are based on period life tables. In other words, they are based on synthetic cohorts which reflect the experience of multiple birth cohorts as they age (e.g., taken in snapshots at 10 or 20-year intervals). There is a necessary confounding of age, period, and cohort effects—and while traditionally demographers make frequent use of age decomposition, there is reason to believe that the trends reported here are driven by cohort effects. Low-educated whites in recent birth cohorts are faring worse than previous birth cohorts with respect to mortality, whereas college-educated whites are faring better now than previous birth cohort. Viewed from a cohort perspective, if this trend continues into the future then mortality will continue to increase as these birth cohort age. I return to this point in the concluding paragraph.

The number of life years lost by cause of death provides a useful measure for comparison within and between education groups, and points to where intervention may be most effective. The difference in temporary life expectancy between ages 25 and 85, _60_*e*_25_, between low- and college-educated white women increased from 2.8 years in 1990 to 7.5 years in 2010. Nearly half of that increase was attributed to smoking-related diseases and external causes of death, where mortality increased dramatically among low-educated white women. The same two causes were responsible for over one third of the 4.1-year increase in the gap in _60_*e*_25_ between low- and college-educated white men. The implication from a health policy perspective is clear—targeting premature deaths from smoking and external causes will have the greatest impact on closing the education gap in life expectancy.

Clearly, cigarette smoking will continue to have a tremendous impact on U.S. adult mortality in the coming decades. Differentials in smoking behavior already explain much of the gender gap in life expectancy [37], the Hispanic mortality advantage [38], and, according to findings here and in recent studies [23], the growing educational gradient in adult life expectancy. Smoking-related cancers and chronic lower respiratory diseases account for a significant loss of life years in all gender and education groups and especially among low and high-school educated men and women. Furthermore, the losses documented in this study necessarily underestimate the full burden of smoking on mortality from heart disease, stroke, and other diseases. The growing number of life years lost to smoking mirrors educational disparities in smoking behavior, which have not only persisted but also widened for whites (and blacks) since the mid-1970s [39].

Perhaps more surprising is the rise in mortality from external causes among all but the college educated. These results, however, are consistent with the recent and dramatic increase in mortality from accidental poisoning, where educational differences are especially large [10,21]. The present study, however, offers several important extensions. First, although several studies documented widening disparities in all-cause and cause-specific mortality by educational attainment, this is the first study to translate those relative risks into actual life years lost in each education group. Second, whereas previous studies suffered from age-aggregation bias [40], this study relied on 5-year abridged life tables to minimize the bias. Indeed, additional analyses reveal that the mean age in each 5-year age group changed by no more than 0.15 years between 1990 and 2010, with no consistent tendency to increase over time across gender-race-education groups aside from the 80–84 age group (results not shown). Third, by using carefully imputed vital statistics data, this study was able to explore trends and disparities in cause-specific mortality across a wider age range than most survey data and among minority groups (results for non-Hispanic blacks are shown in Supporting Information, S3 Appendix).

This study is not without limitations. Education misreporting and missing information in the vital registry data are a potential source of bias [17], as is the change in underlying cause of death classification during the study period. Several steps were taken to minimize both random and systematic sources of error [13], but they cannot be eliminated with certainty. Grouping causes of death in nine major categories ensures consistency over time, but comes at the expense of a more detailed analysis. It is nevertheless reassuring that results in this study are consistent with prior research based on the National Health Interview Survey, both with respect to the direction and magnitude of changes in age- and cause-specific mortality [8,22].

Finally, adopting a similar approach to multiple other studies [41–44], this study estimated the gross association between education and longevity and how that association had changed over time. However, adult mortality is affected by a multitude of social, economic, behavioral, and environmental determinants—some, but not all, of which are caused by or associated with educational attainment. Educational attainment serves a convenient indicator of socioeconomic status because it occurs early in the life course and remains stable thereafter. Furthermore, it shapes much of one's life course trajectory in a variety of ways. Indeed, the effect of education on adult mortality may operate via income [45], health behaviors [46], marriage [47,48], chronic stress [49], or their combination. At the same time, family background is known to determine both academic achievement [50] and adult mortality [51] which may render part of this association spurious. The extent to which the relationship between education and adult mortality is causal remains contested [52,53] and deserves further scrutiny in order to design effective policy interventions.

Nevertheless, it appears that large segments of the U.S. population no longer partake in the remarkable decline in mortality that characterized much of the 20^th^ century. Indeed, some groups have even been regressing, as was previously reported for low-educated whites—those with fewer than 12 years of schooling—and more recently even among the high-school educated in midlife [7,10,11–13]. The results in this study further highlight that while old-age mortality declined among high school—educated whites, the subsequent gains in $e_{25}^{o}$ were partially, if not entirely, offset by increasing mortality under age 55. It is difficult to determine whether these patterns reflect period or cohort trends because they are based on period life tables, which conjoin multiple birth cohorts into a single, synthetic cohort. Since the 1960s, however, changes in mortality from heart disease and lung cancer in the U.S. have largely been driven by cohort effects [54], which also explain the growing educational differences in mortality from those causes [55]. If the same is true for other causes of death, then the rise in young-adult and midlife mortality may be a precursor to what lies ahead for those groups as they enter old age.

## Supporting Information

S1 Appendix ICD codes used for cause of death grouping. (PDF)

S2 Appendix Midyear population estimates and number of deaths by gender, race, education, and cause. (PDF)

S3 Appendix Results for non-Hispanic blacks. (PDF)
