# Supplementary material for: Diverging Trends in Cause-Specific Mortality and Life Years Lost by Educational Attainment: Evidence from United States Vital Statistics Data, 1990-2010
Source: PLoS One. 2016 Oct 4;11(10):e0163412. doi: 10.1371/journal.pone.0163412 (PMC5049791; doi:10.1371/journal.pone.0163412)
Supplement: S1 Appendix — (PDF) [file pone.0163412.s001.pdf]

S1 Appendix. ICD codes used for cause of death grouping.

Cause of death	ICD-9	ICD-10
Infectious and parasitic diseases	0-139	A00-B99
Neoplasms (excluding smoking related)	140-149, 151-160, 163-239	C16-C31, C35-D48
Cardiovascular diseases	390-429, 440-459	I00-I59, I70-I99
Cerebrovascular diseases	430-438	I60-I69
Respiratory diseases (excluding smoking-related)	460-489, 493-495, 497-519	J00-J39, J45-J98
Smoking-related diseases (cancers of the lip, oral cavity, pharynx, esophagus, larynx, trachea, lung, and bronchus; bronchitis, emphysema, and chronic airway obstruction)	150, 161-162, 490-492, 496	C00-C15, C32-C34, J40-J44
Diabetes mellitus	250	E10-E14
External	E800-E999	V01-Y89
Other	All remaining codes	All remaining codes

Notes: Deaths in 1990 are classified under ICD-9 codes whereas deaths in 2000 and 2010 are classified under ICD-10; Smoking-related diseases include all causes where the smoking attributable fraction of deaths exceeds 65 percent in both genders combined (see main text for details).
