# Supplementary material for: Diverging Trends in Cause-Specific Mortality and Life Years Lost by Educational Attainment: Evidence from United States Vital Statistics Data, 1990-2010
Source: PLoS One. 2016 Oct 4;11(10):e0163412. doi: 10.1371/journal.pone.0163412 (PMC5049791; doi:10.1371/journal.pone.0163412)
Supplement: S2 Appendix — (PDF) [file pone.0163412.s002.pdf]

S2 Appendix. Midyear population estimates and number of deaths by gender, race, education, and cause.

Table S2.1: Number of deaths by gender, education, and cause among non-Hispanic whites 1990-2010.

Gender	Year	Education (years)	Midyear population	Infectious & parasitic	Neoplasms	Cardio-vascular	Respiratory	External	SRD*	Cerebro-vascular	Diabetes	Other	Total
**Women**	**1990**	**0-11**	12,070,975	4,782	50,393	145,973	21,718	7,958	26,499	31,846	9,133	40,456	**338,758**
		**12**	29,660,127	4,809	68,766	126,089	18,395	11,120	35,536	27,089	7,899	39,059	**338,762**
		**13-15**	11,799,518	1,255	20,754	34,171	5,412	4,170	9,587	8,461	1,950	11,814	**97,574**
		**16+**	12,414,257	1,146	17,701	27,371	4,524	3,361	6,697	6,847	1,385	9,663	**78,695**
	**2000**	**0-11**	8,281,916	5,902	39,035	116,830	17,735	7,626	34,329	28,827	9,577	54,844	**314,705**
		**12**	29,702,519	8,157	76,638	144,219	22,043	13,408	58,494	36,431	11,917	72,190	**443,497**
		**13-15**	14,961,916	2,278	26,046	41,407	6,852	5,491	17,117	11,594	3,223	23,125	**137,133**
		**16+**	17,494,138	1,719	23,237	30,820	5,493	4,473	11,070	9,309	1,946	18,350	**106,417**
	**2010**	**0-11**	5,571,690	4,994	22,964	59,864	9,687	6,951	27,484	13,252	5,393	53,040	**203,629**
		**12**	27,669,555	11,890	75,870	130,562	20,968	20,044	68,986	30,703	11,503	120,234	**490,760**
		**13-15**	17,318,233	3,854	31,207	40,028	6,826	9,725	22,769	10,238	3,551	40,125	**168,323**
		**16+**	22,305,450	2,609	28,311	28,288	5,018	6,573	13,619	7,570	2,125	30,183	**124,296**
**Men**	**1990**	**0-11**	10,347,168	5,286	50,007	136,718	19,789	16,708	53,952	20,322	6,210	33,682	**342,674**
		**12**	23,733,128	8,218	51,194	117,884	13,826	24,346	47,453	15,230	5,784	29,985	**313,920**
		**13-15**	10,361,470	4,235	17,102	35,240	4,148	8,210	13,288	4,758	1,792	9,707	**98,480**
		**16+**	15,252,000	5,508	22,584	42,340	5,372	7,961	13,502	5,886	1,853	11,702	**116,708**
	**2000**	**0-11**	7,540,518	5,041	38,290	95,180	14,343	13,861	48,120	16,734	6,977	35,732	**274,278**
		**12**	24,812,189	7,667	58,808	123,255	16,229	26,438	59,573	19,402	9,483	46,900	**367,755**
		**13-15**	13,467,696	2,742	22,388	40,897	5,343	9,745	18,825	6,838	3,376	16,803	**126,957**
		**16+**	18,946,760	3,083	30,758	49,345	7,071	9,526	18,590	8,944	3,572	20,957	**151,846**
	**2010**	**0-11**	5,605,093	4,593	25,079	53,984	9,052	12,273	34,513	8,202	5,104	34,352	**187,152**
		**12**	25,232,736	10,142	64,643	115,606	16,887	36,526	68,025	16,470	11,093	72,261	**411,653**
		**13-15**	15,233,235	4,006	29,693	45,122	6,529	15,551	24,563	6,743	4,517	29,304	**166,028**
		**16+**	22,063,609	3,957	38,765	51,322	8,352	13,381	21,156	8,546	4,370	36,884	**186,733**

Notes: Number of deaths averaged over ten imputations; *SRD = smoking-related diseases (bronchitis, emphysema, chronic airway obstruction and cancers of the lip, oral cavity, pharynx, esophagus, larynx, trachea, lung, and bronchus).

Table S2.2: Number of deaths by gender, education, and cause among non-Hispanic blacks 1990-2010.

Gender	Year	Education (years)	Midyear population	Infectious & parasitic	Neoplasms	Cardio-vascular	Respiratory	External	SRD*	Cerebro-vascular	Diabetes	Other	Total
**Women**	**1990**	**0-11**	2,687,518	1,878	9,628	24,323	2,814	1,707	3,278	5,824	2,876	8,287	**60,615**
		**12**	3,802,307	1,543	6,744	11,704	1,476	1,812	2,400	2,619	1,530	4,778	**34,606**
		**13-15**	1,503,842	416	1,966	2,554	370	589	555	626	327	1,180	**8,583**
		**16+**	1,067,580	256	1,579	2,213	293	334	378	578	266	950	**6,847**
	**2000**	**0-11**	2,195,391	2,586	8,190	21,489	2,591	1,388	3,974	5,467	3,372	9,906	**58,963**
		**12**	4,640,971	2,628	8,738	15,891	1,929	1,822	4,319	3,702	2,636	7,450	**49,115**
		**13-15**	2,442,581	735	3,196	4,362	564	728	1,258	1,098	711	2,219	**14,871**
		**16+**	1,676,881	390	2,411	3,120	415	382	814	821	477	1,630	**10,460**
	**2010**	**0-11**	1,822,174	1,891	5,971	12,916	1,672	1,008	3,473	3,083	2,098	10,581	**42,693**
		**12**	4,810,855	2,610	10,163	15,832	2,100	1,962	5,482	3,586	2,708	11,335	**55,778**
		**13-15**	3,595,970	968	5,143	5,895	778	1,094	2,144	1,340	1,099	4,304	**22,765**
		**16+**	2,532,915	476	3,250	3,419	472	520	1,083	895	511	2,859	**13,485**
**Men**	**1990**	**0-11**	2,248,211	2,974	11,145	23,329	3,426	5,324	9,412	4,723	1,650	8,717	**70,700**
		**12**	3,174,672	3,466	5,708	11,385	1,680	6,068	4,620	2,004	959	5,279	**41,169**
		**13-15**	1,097,340	1,236	1,328	2,680	360	1,572	950	452	227	1,281	**10,086**
		**16+**	808,280	834	1,110	1,843	272	697	620	337	166	822	**6,701**
	**2000**	**0-11**	1,974,633	3,115	8,822	17,762	2,483	3,735	8,170	3,977	1,982	7,693	**57,739**
		**12**	4,089,879	4,013	7,533	15,088	1,819	5,760	6,399	2,772	1,855	6,993	**52,232**
		**13-15**	1,840,865	1,057	2,081	3,914	430	1,671	1,431	691	507	1,806	**13,588**
		**16+**	1,197,480	659	1,716	2,818	306	718	888	500	370	1,156	**9,131**
	**2010**	**0-11**	1,799,402	1,970	6,580	11,964	1,685	2,916	5,887	2,323	1,682	7,453	**42,460**
		**12**	4,668,822	3,399	9,675	17,271	1,958	6,263	7,511	3,015	2,486	9,401	**60,979**
		**13-15**	2,617,390	1,074	3,487	5,679	575	2,063	2,109	937	899	3,034	**19,857**
		**16+**	1,691,059	544	2,311	3,363	377	785	1,009	584	512	1,816	**11,301**

Notes: Number of deaths averaged over ten imputations; *SRD = smoking-related diseases (bronchitis, emphysema, chronic airway obstruction and cancers of the lip, oral cavity, pharynx, esophagus, larynx, trachea, lung, and bronchus).
