# Supplementary material for: Diverging Trends in Cause-Specific Mortality and Life Years Lost by Educational Attainment: Evidence from United States Vital Statistics Data, 1990-2010
Source: PLoS One. 2016 Oct 4;11(10):e0163412. doi: 10.1371/journal.pone.0163412 (PMC5049791; doi:10.1371/journal.pone.0163412)
Supplement: S3 Appendix — (PDF) [file pone.0163412.s003.pdf]

S3 Appendix. Results for non-Hispanic blacks.

Fig S3.1. Age decomposition of change in life expectancy by gender and years of schooling, non-Hispanic blacks 1990-2010.

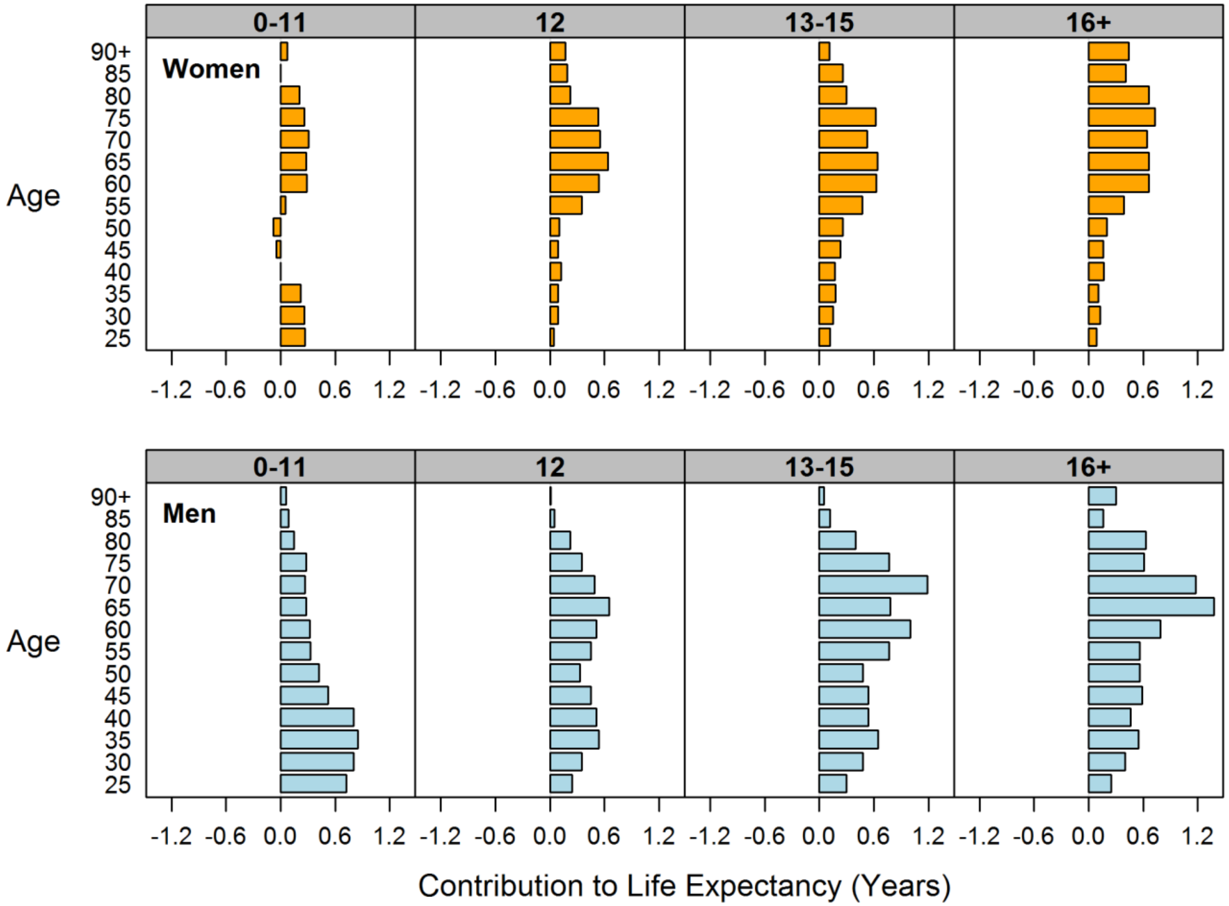

Fig S3.2. Total life years lost between ages 25 and 85 by gender and years of schooling, non-Hispanic blacks 1990-2010.

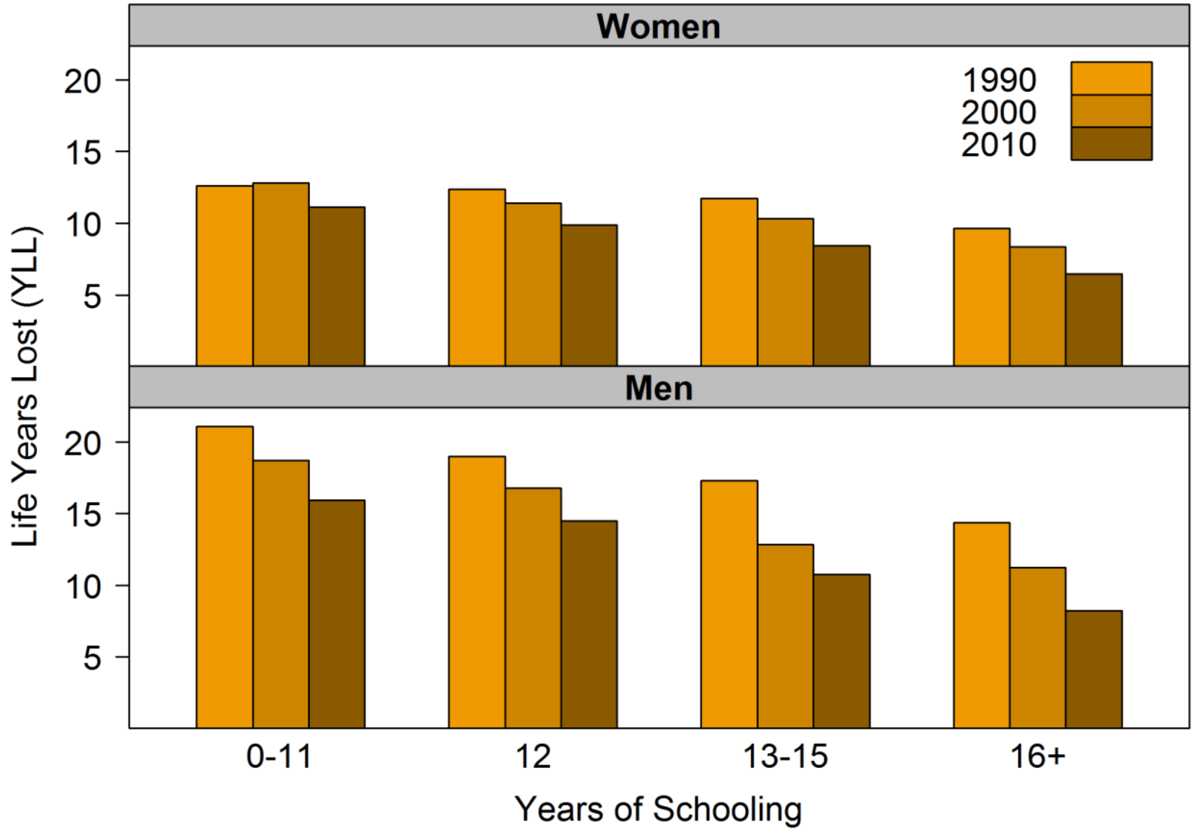

Fig S3.3. Life years lost between ages 25 and 85 by cause of death and years of schooling, non-Hispanic black women.

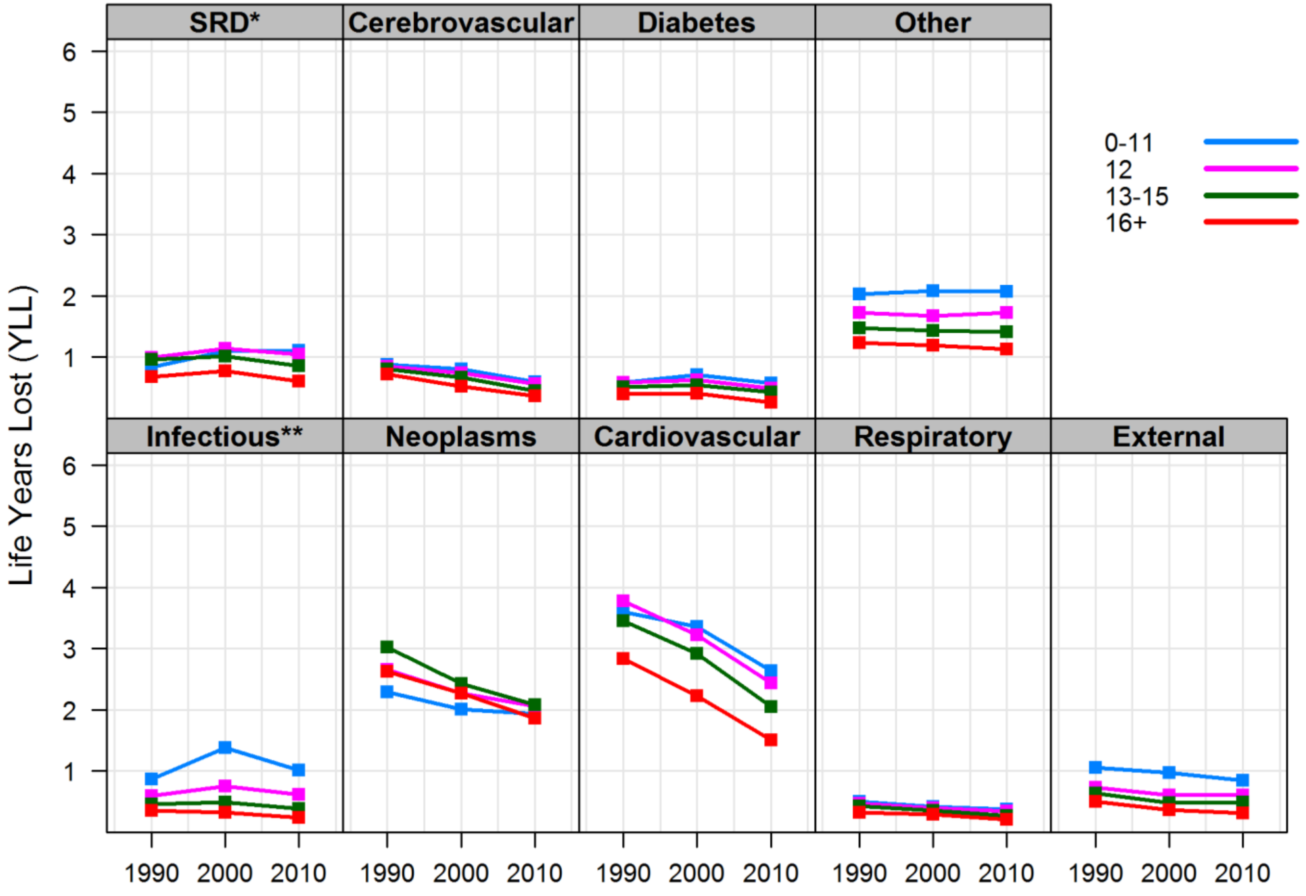

Note: * SRD = smoking-related diseases (bronchitis, emphysema, chronic airway obstruction and cancers of the lip, oral cavity, pharynx, esophagus, larynx, trachea, lung, and bronchus); ** Infectious and parasitic diseases.

Fig S3.4. Life years lost between ages 25 and 85 by cause of death and years of schooling, non-Hispanic black men.

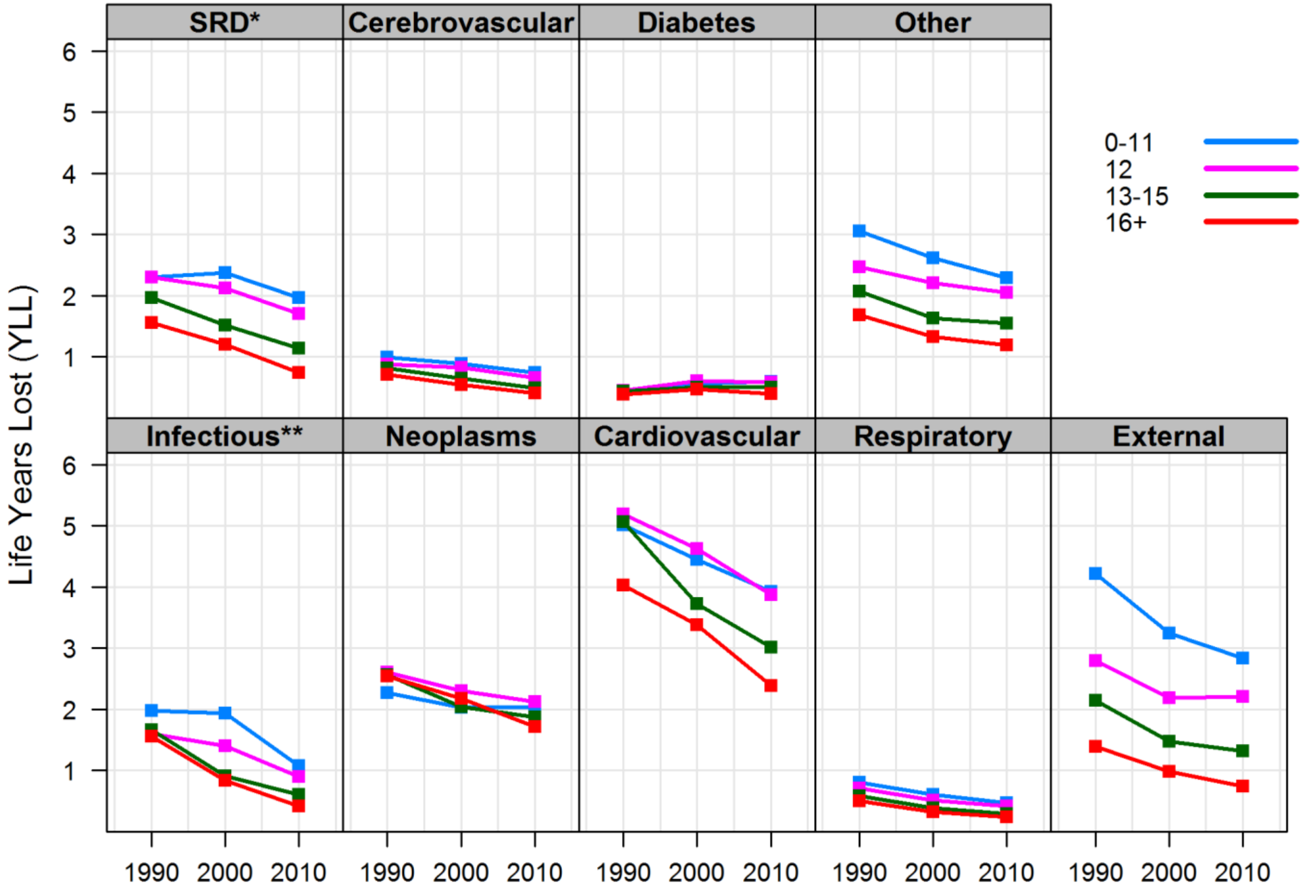

Note: * SRD = smoking-related diseases (bronchitis, emphysema, chronic airway obstruction and cancers of the lip, oral cavity, pharynx, esophagus, larynx, trachea, lung, and bronchus); ** Infectious and parasitic diseases.
